# Special Issue “In Vivo Nuclear Molecular Imaging in Drug Development and Pharmacological Research”

**DOI:** 10.3390/ph16030459

**Published:** 2023-03-20

**Authors:** Xuyi Yue

**Affiliations:** 1 Diagnostic & Research PET/MR Center, Nemours Children’s Health, Wilmington, DE 19803, USA; xuyi.yue@nemours.org; 2 Department of Radiology, Nemours Children’s Health, Wilmington, DE 19803, USA

Nuclear molecular imaging is increasingly important in aiding diagnosis, monitoring disease progression, and assessing response to treatment. It is an essential tool in drug development and pharmacological research to study mechanisms of action, identify targets, evaluate receptor occupancy, determine dose regimens, and investigate pharmacokinetic and pharmacodynamic properties. In recent years, the United States Food and Drug Administration (FDA) approved several radioactive diagnostic agents, including piflufolastat F-18 ([^18^F]DCFPyL), gallium 68 PSMA-11, gallium Ga 68 gozetotide, and Fluorodopa F18 injection, for prostate cancer and suspected Parkinsonian syndromes, highlighting the critical and complementary role of nuclear molecular imaging in addition to traditional imaging modalities.

Our call for papers for this Special Issue, In Vivo Nuclear Molecular Imaging in Drug Development and Pharmacological Research, received great interest from a broad range of researchers from different fields. We have published 12 papers, involving approximately 100 authors from nine countries. We are delighted to see that the research includes a range of fields, from basic science studies to clinical translation investigations. This reprint describes cutting-edge research from a diverse community.

*N*-methyl-d-aspartate receptors (NMDAR) play a pivotal function in neurodegenerative diseases. However, the therapeutics targeting NMDA receptor subunits GluN1/2B need to be improved due to the lack of a selective radioligand for drug screening. To circumvent the limitations of the most commonly used but unselective [^3^H]ifenprodil for GluN1/2B competitive binding assay, Ahmed et al. developed a tritiated version of OF-NB1 [1]. This research is a continuation of the team’s previous exciting work [2,3,4,5]. [^3^H]OF-NB1 showed good selectivity over the sigma 1 receptor. Furthermore, in vitro binding assay of the known GluN1/2B antagonist and sigma one compound with [^3^H]OF-NB1 and [^3^H]ifenprodil and in vivo receptor occupancy study in rats validate the favorable profile of [^3^H]OF-NB1.

Prostate-specific membrane antigen (PSMA) is expressed in more than 90% of prostate cancer patients [6]. Unfortunately, several reported PSMA radiotracers either show unfavorable kinetics or high uptake in non-target organs. Basuli et al. developed a series of fluorine-18-labeled oxime radiotracers based on the reported Lys-Urea-Glu scaffold by modulating the lipophilicity. The most lipophilic radiotracer maintained good in vitro binding affinity, a high tumor-to-non-target ratio in vivo, and comparable tumor uptake when compared with the FDA-approved [^18^F]DCFPyL. Furthermore, the simple structural modification significantly lowered the kidney uptake, which may provide a strategy to reduce nephrotoxicity [7].

Marie et al. used FDA-approved ^99m^Tc-mebrofenin to evaluate hepatocyte transporter function. The dysregulation of hepatocyte transporters is closely associated with impaired liver function and hepatotoxicity. This study found that lipopolysaccharide (LPS)-treated rats showed a dramatic downregulation of hepatocyte transporters, including multidrug resistance-associated proteins 2 and 3. Interestingly, the antituberculosis drug rifampicin, a potent inhibitor of hepatocyte transporters, showed very different effects on the hepatocyte transporters in both control and LPS-treated rats. ^99m^Tc-mebrofenin imaging may show potential in precision medicine with optimized dose selection for various drugs [8].

Microvascular disease is frequently associated with major pathologies, including atherosclerosis, diabetes, dementia, and stroke. It can occur in several vital organs, such as the brain, heart, and kidneys [9]. Existing strategies to non-invasively detect microvascular disease are limited. Wang et al. successfully used ^18^F-fluorodeoxyglucose (^18^F-FDG)-labeled rat red blood cells (^18^F-FDG RBC) to map brain total vascular volume and intramyocardial vascular volume changes in rats challenged by a coronary artery vasodilator. In addition, ^18^F-FDG-labeled erythrocytes can localize infarcted myocardium in a myocardial infarction rat model. The results correlate with metabolic ^18^F-FDG positron emission tomography (PET) imaging and were further validated by tissue staining. Furthermore, ^18^F-FDG RBC PET can map drug-induced intra-myocardial vasodilation in diabetic rats and normal controls [10]. This technique is operationally simple and may be promising in the non-invasive detection of whole-body microvascular pathologies and evaluation of treatment response with therapeutics targeting microvascular diseases.

Mesenchymal stem cell-derived extracellular vesicles (MSC-EV) therapy is promising as a treatment for type 1 and type 2 diabetes due to its efficiency in transferring serial biological molecules to modulate immune responses and metabolic functions. Therefore, the safe delivery and tracking of MSC-EVs are critical for diabetes therapies. Li et al. developed iodine-124-labeled umbilical cord MSC-EV, which showed over 95% purity over 4 h. The researchers used two administration routes (intra-arterial vs. intravenous) to conduct a pilot study in non-diabetic Lewis rats to guide iodine-124-labeled umbilical cord MSC-EV delivery. The results show that the two strategies display similar delivery efficacies, except in the spleen and liver. However, the intravenous administration method is preferred since it is much less invasive and operationally simple compared with the invasive and challenging intra-arterial delivery [11].

Chen et al. reported a heterobivalent peptide modified with thin layer-protected gold nanoparticles for multiple imaging of esophageal cancer in a human xenograft model. The nanoprobe features good stability and biocompatibility, dual targeting of epidermal growth factor receptors and erb-b2 receptor tyrosine kinase 2, multimodal imaging with photoacoustic and computed tomography, and favorable in vivo kinetics. In addition, the dual targeting strategy shows promise for detecting cancers in the early stages due to improved sensitivity [12].

Alzheimer’s disease (AD) is the leading cause of dementia. The recent failure of crenezumab, an investigational anti-amyloid drug, is the latest setback in effective AD treatment [13]. Therefore, preclinical animal models with various PET probes are critical to research mechanisms of action and develop potential therapeutics. Ni contributed to a comprehensive review of AD imaging in several animal models with PET modality. The author reviewed well-studied biomarkers, including amyloid, brain glucose metabolism, and synaptic and neurotransmitter receptors, and discussed new biomarkers in AD, such as microtubule and mitochondria imaging. The author also addressed the challenges of translating the rodent AD model to a clinical investigation and proposed models close to human AD pathology [14].

On 23 March 2022, the FDA approved gallium Ga 68 gozetotide injection, a peptide conjugate, for the diagnosis of PSMA-positive lesions in males with prostate cancer. On the same day, the FDA approved the amino acid-based Lutetium (^177^Lu) vipivotide tetraxetan for treating patients with castration-resistant prostate cancer [15]. Radiometal-based agents have received increasing attention due to favorable half-life, easily adaptable clinical production, and radiotheranostics implementation. New radionuclides that can easily be distributed to satellite sites, have favorable positron emission energies, and are operationally simple for a therapeutic match to meet personalized medicine requirements are still attractive. Fonseca et al. reported two routes to produce clinical doses of ^61^Cu-based radiopharmaceuticals with fully automated Good Manufacturing Practice (GMP)-compliant procedures. The purity of the two targets significantly affects the yield of copper-61. The utilization of the produced copper-61 was demonstrated by a fully automated GMP-compliant production of three radiopharmaceuticals labeled with gallium-68 in clinical practice. Copper-61 may serve as an alternative radionuclide to the widely used gallium-68 [16].

Fibroblast activation protein (FAP) is a novel target for the molecular imaging of oncology and cardiovascular disease [17,18]. The research by Diekmann et al. using ^68^Ga-fibroblast-activation protein-46 (^68^Ga-FAP-46) PET/CT to predict myocardial infarction was selected as the Society of Nuclear Medicine and Molecular Imaging (SNMMI) Image of the Year 2022 [19,20]. In this Special Issue of *Pharmaceuticals*, Vallejo-Armenta et al. reported the findings of a boronic acid derivative, [^99m^Tc]Tc-{(*R*)-1-[(6-hydrazinylnicotinoyl)-d-alanyl]pyrrolidin-2-yl}-labeled boronic acid ([^99m^Tc]Tc-iFAP), as the radioligand targeting FAP in 32 patients with six different cancer entities. The results show that [^99m^Tc]Tc-iFAP can effectively detect high-grade World Health Organization (WHO) III–IV gliomas with a 100% sensitivity for primary tumors, while it is inferior to ^18^F-FDG in lymph node metastases and distant metastases cases. However, patients with peritoneal carcinomatosis lesions in recurrent colorectal cancer show only [^99m^Tc]Tc-iFAP uptake, demonstrating its valuable complementary role for prognostic evaluation [21].

Son et al. reviewed PET/MR hybrid systems and their applications in psychiatric disorders. The authors discussed the advancements of PET, MRI, and fusion PET-MRI technology in clinical settings. While there are many carbon-11 and fluorine-18-labeled tracers targeting serotonin receptors and transporters, glucose, dopamine receptors, and phosphodiesterase 10A, etc., for clinical investigations, the authors stated that improving the PET spatial resolution to match MRI is crucial for reliable and quantitative analysis. The team achieved a 1.56 mm full width at the half-maximum transaxial resolution, a resolution even higher than the high-resolution research tomograph (2.47 mm) [22].

Netufo et al. reviewed intraoperative fluorescence imaging agents for guiding glioblastoma surgery in preclinical research and clinical practice. 5-Aminolevulinic acid (5-ALA) is the only FDA-approved intra-operative fluorescence imaging agent for glioblastoma patients. However, 5-ALA has several limitations, including challenges in identifying critical neurological components under dark-field conditions, photobleaching, and 2D-only images. Therefore, the authors emphasize the importance of using a targeting strategy and a combination of multimodal imaging, such as PET-guide surgical planning with intra-operative fluorescence imaging agents, to determine the extent of resection and improve overall survival [23].

Lung ventilation–perfusion scintigraphy is a critical technique to assess regional ventilation and perfusion function. Currently, most radiopharmaceuticals for lung function are technetium-99m (^99m^Tc)-based single-photon emission computerized tomography (SPECT) agents. However, PET has a higher sensitivity, resolution, and better quantitative capacity than SPECT. Therefore, Blanc-Béguin et al. reviewed the chemical, technical, and pharmacological aspects of ^99m^Tc- and ^68^Ga-based lung ventilation and perfusion imaging agents and discussed the advantages and challenges of transition from ^99m^Tc- to ^68^Ga-labeled agents for optimal clinical use. The authors concluded that minimal pharmacological property changes and simplified and GMP-compliant automated procedures are essential for switching from ^99m^Tc- to ^68^Ga-labeled nuclear imaging agents for lung functions [24].

In summary, this Special Issue highlights the opportunities and challenges in nuclear molecular imaging from preclinical research to clinical translation and covers a broad overview of the field. I sincerely thank all the authors for their valuable contributions to this Special Issue. I also thank all the reviewers and editors for their tremendous support. I hope the articles and reviews in this Special Issue meet readers’ expectations in the field and further promote nuclear molecular imaging research in the community.

## Data Availability

Data is contained within the article.
